# Association between Sarcopenia and Balance in Patients Undergoing Inpatient Rehabilitation after Hip Fractures: A Retrospective Cohort Study

**DOI:** 10.3390/medicina60050742

**Published:** 2024-04-29

**Authors:** Ryu Ishimoto, Hirotaka Mutsuzaki, Yukiyo Shimizu, Ryoko Takeuchi, Shuji Matsumoto, Yasushi Hada

**Affiliations:** 1 Graduate School of Comprehensive Human Sciences, University of Tsukuba, Tsukuba 305-8575, Japan; ishimotori@ipu.ac.jp; 2 Department of Rehabilitation Medicine, Ibaraki Prefectural University of Health Sciences Hospital, Ami 300-0331, Japan; 3 Center for Medical Science, Ibaraki Prefectural University of Health Sciences, Ami 300-0394, Japan; 4 Department of Orthopaedic Surgery, Ibaraki Prefectural University of Health Sciences Hospital, Ami 300-0331, Japan; 5 Department of Rehabilitation Medicine, Institute of Medicine, University of Tsukuba, Tsukuba 305-8575, Japan

**Keywords:** balance, Berg Balance Scale, hip fractures, inpatient rehabilitation, sarcopenia

## Abstract

*Background and Objectives:* Sarcopenia is characterized by a decline in skeletal muscle mass, strength, and function and is associated with advancing age. This condition has been suggested as a factor that negatively influences the functional outcomes of patients with hip fractures. However, the association between sarcopenia and balance impairment in patients undergoing inpatient rehabilitation after hip fractures remains unclear. In this retrospective cohort study, we aimed to investigate the impact of sarcopenia on balance outcomes in patients undergoing inpatient rehabilitation following hip fractures. *Materials and Methods:* Baseline sarcopenia was diagnosed using skeletal muscle mass index and handgrip strength, with cut-off values recommended by the Asian Working Group for Sarcopenia. The primary outcome was balance, which was assessed using the Berg Balance Scale (BBS) at the time of discharge. A multiple linear regression model analyzed the association between sarcopenia and balance. The model was adjusted for age, sex, comorbidities, and cognitive function. *Results:* Among the 62 patients (mean age: 78.2; sex: 75.8% women), 24.2% had sarcopenia. Patients with sarcopenia had significantly lower BBS scores than did those without sarcopenia (41 vs. 49 points, *p* = 0.004). Multiple linear regression analysis revealed that baseline sarcopenia was independently associated with BBS scores at discharge (β = −0.282, *p* = 0.038). *Conclusions:* Following inpatient rehabilitation, patients with baseline sarcopenia had inferior balance outcomes than did those without sarcopenia at discharge. Sarcopenia should be assessed on admission to consider and provide additional care for those with a higher risk of poor functional outcomes. More studies are needed to investigate the association between sarcopenia and functional outcomes, examine the impact of sarcopenia treatment on these outcomes, and reduce the risk of recurrent falls and fractures in patients with hip fractures.

## 1. Introduction

The incidence of hip fractures is rising in Asia because of the increasing aging population. Projections indicate that this incidence will escalate from approximately 1.1 million in 2018 to 2.6 million by 2050, with medical costs reaching U.S. $15 billion [1]. These fractures pose significant challenges, resulting in high mortality rates, long-term morbidity, functional dependence, decreased quality of life, and socioeconomic burden [2,3,4,5]. As the burden of care increases following a fracture, recognizing risk factors associated with functional outcomes and improving the quality of care and rehabilitation after a hip fracture are crucial for maximizing activities of daily living (ADLs) and preventing secondary falls and fractures [1].

Several risk factors are associated with functional outcomes after hip fracture. Advancing age, pre-existing mobility issues, postoperative complications, and decreased cognition are associated with poor functional outcomes in hip fractures [6,7,8,9]. Several studies have recognized sarcopenia as a factor that negatively influences functional outcomes in patients with hip fractures [10,11,12,13,14,15,16]. Sarcopenia is characterized by a decline in skeletal muscle mass, strength, and function accompanied by advancing age [17]. These consequences are detrimental because sarcopenia is associated with unfavorable outcomes, such as an increased risk of falls and fractures, limited physical function, institutionalization, and mortality [11,18,19,20,21]. A high prevalence of sarcopenia has been reported in patients with hip fractures, with estimates ranging from 17% to 61% depending on factors such as the study population, the diagnostic tools employed, and the definition of sarcopenia utilized [12,14,15,22,23].

Improving physical function and independence following surgery is imperative, alongside taking proactive measures to prevent the recurrence of falls. However, data on the relationship between sarcopenia and functional outcomes in patients undergoing inpatient rehabilitation for hip fractures are scarce [11,14]. Additionally, studies investigating this association have not fully explored the balance. Elderly individuals who have undergone hip fracture surgery often struggle with balance problems, which may impede their ADLs and ability to move, thereby increasing their risk of falls [24,25].

Therefore, in this study, we aimed to investigate the impacts of sarcopenia on the outcome of balance in patients undergoing inpatient rehabilitation for hip fracture. Based on the findings of previous studies [11,12,13,15], we hypothesized that patients with baseline sarcopenia would exhibit inferior balance outcomes compared with those of patients without sarcopenia at the time of discharge.

## 2. Materials and Methods

### 2.1. Participants and Setting

This study was conducted at Ibaraki Prefectural University of Health Sciences Hospital, a rehabilitation hospital with 120 beds. Patients were referred to our hospital for subacute care and inpatient rehabilitation. Eligible patients were diagnosed with unilateral femoral neck, intertrochanteric, or subtrochanteric fractures, and admitted to our hospital for postoperative inpatient rehabilitation between May 2017 and July 2023. Patients with acute to subacute bilateral or multiple lower limb fractures, those transferred to other hospitals owing to acute medical conditions, and those with missing data were excluded from the study.

**Figure 1:**
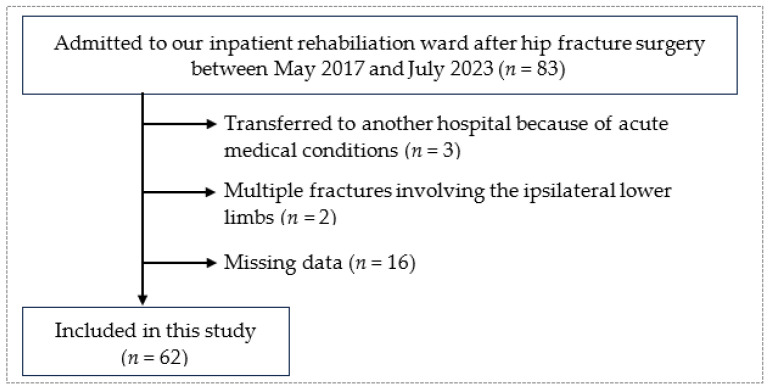
Recruitment flowchart.

The recruitment flowchart is shown in Figure 1. Overall, 83 patients were referred to our hospital during the study period. Three patients were transferred to other hospitals due to one of the following medical conditions: acute cholecystitis, acute heart failure, and symptomatic chronic epidural hematoma. Two patients sustained subacute multiple fractures of the ipsilateral lower limbs. Sixteen patients were missing baseline data. The analysis excluded these patients, while the remaining 62 patients were included in this study. 

The rehabilitation program comprised individualized physical and occupational therapies based on the functional abilities of each patient. The program included a joint range of motion exercises, muscle strengthening, balance exercises, gait training, stair training, endurance exercises, and training for ADLs. The rehabilitation program was planned for 120–180 min/day, 6–7 days a week. Moreover, the program included patient education and personalized nutritional management provided by registered dietitians.

### 2.2. Study Design and Data Collection

This retrospective cohort study examined the impact of baseline sarcopenia on balance in patients who underwent inpatient rehabilitation for hip fracture. Baseline data, including age, sex, cause of injury, fracture type, surgery type, surgery-to-dual energy X-ray absorptiometry (DXA)-scan duration, comorbidities, pre-fracture walking ability, and ADLs on admission, were collected through chart extraction. The severity of comorbidities was assessed using the original version of the Charlson Comorbidity Index (CCI) [26]. ADLs were assessed using the Functional Independence Measure (FIM) [27,28]. Height and weight were measured on admission using measuring tape and electronic scales, respectively. The body mass index (BMI) was calculated as body weight divided by the square of body height (kg/m^2^) [29]. The handgrip strength of the dominant hand was measured using a hand dynamometer. In patients with hemiparesis, the non-paralyzed hand was used for assessment. Appendicular skeletal muscle mass (ASM) was measured using whole-body DXA with a Horizon A model (Hologic Inc., Marlborough, MA, USA). APEX software version 5.6.04 (Hologic Inc.) was used to acquire and analyze the whole-body scans.

### 2.3. Outcome Measurements

The primary outcome was balance, which was assessed using the Japanese version of the Berg Balance Scale (BBS) at the time of discharge [30]. The BBS evaluates balance in various postures and movements [31]. It includes 14 tasks, including standing up, sitting down, standing independently, standing with eyes closed, standing with the upper arm extended, turning around, taking alternating steps with both feet, and standing on one leg. Each task is scored on a 5-point scale (0–4 points), and the total possible score is 56 points. Lower scores indicated poorer balance and an increased risk of falls. The BBS has been demonstrated to have high reliability and validity in older adults [32,33]. Patients were stratified into the good and poor balance groups based on the cutoff value suggested for fall risk (BBS > 45 points) [31].

The secondary outcomes were ADLs, cognitive function, and walking ability. ADLs and cognitive function were assessed using the Japanese version of FIM, version 3.0, which is a widely used assessment tool in the motor and cognitive domains [27,28]. It includes 13 motor tasks, including self-care, sphincter control, transfers, and locomotion tasks; and five cognitive tasks, including communication and social cognition tasks. Each task is scored on a 7-point scale (1–7 points) that represents the degree of assistance from total dependence to complete independence. The total score ranges from 18 to 126 points, with the FIM-motor score ranging from 13 to 91 points and the FIM-cognition score ranging from 5 to 35 points. Lower scores indicate higher levels of dependency.

Walking ability was assessed using the original version of the Functional Ambulation Category (FAC) and self-selected walking speed (SWS). The FAC assesses a patient’s ability to walk independently, indoors, outdoors, and on stairs [34]. The scale classifies the need for walking assistance into six categories (score of 0–5 points), with higher scores indicating superior walking ability. The FAC scale has high test-retest and inter-rater reliability for individuals undergoing inpatient rehabilitation [35]. The FAC was initially developed to evaluate walking ability in patients with hemiplegia; however, it has also been used in patients with hip fractures [7,36,37]. The participants were classified as independent (FAC > 3) or dependent ambulators (those requiring physical assistance) (FAC ≤ 3) in accordance with a previous study [37]. The SWS was determined based on the time required to walk a 10-m distance. The participants walked a 16-m distance, and the time taken to cover the intermediate 10-m distance was measured to allow for acceleration and deceleration. The measurement was repeated up to a maximum of three times, and the fastest time was recorded for analysis.

### 2.4. Diagnosis of Sarcopenia

Patients were diagnosed as having sarcopenia when low skeletal muscle mass index (SMI) and decreased handgrip strength were observed, in accordance with the criteria recommended by the Asian Working Group for Sarcopenia (AWGS) [17]. The use of BMI-adjusted SMI is suggested by the AWGS when assessing ASM using DXA [17]. Additionally, a study reported that BMI-adjusted SMI was more strongly associated with low muscle strength and function than was height-adjusted SMI (adjusted by the square of body height) in the elderly [38]. Therefore, in this study, the SMI was determined by dividing the ASM by the BMI. The SMI cut-off values for men and women were <0.789 and <0.512 kg/kg/m^2^, respectively. The cut-off values for handgrip strength for men and women were <28 kg and <18 kg, respectively.

### 2.5. Statistical Analysis

Data are presented in one of three formats: numerical values (%) for categorical data, means (standard deviations) for parametric data, or medians (interquartile ranges) for nonparametric data. The normality of the data was assessed using the Shapiro–Wilk test, while the equality of variance was evaluated using Levene’s test. Depending on the variables, the chi-square test, Fisher’s exact test, Student’s *t*-test, and the Mann–Whitney U test were used to examine and compare the baseline characteristics of the participants between the groups with and without sarcopenia. Multiple linear regression analysis was conducted to examine the association between baseline sarcopenia and BBS scores assessed at discharge. Based on the findings of previous studies [16,39,40], the model was adjusted for participant characteristics, including age, sex, comorbidity (assessed using the CCI), and cognitive function (assessed using the FIM cognition score). The variance inflation factor (VIF) was used to evaluate the presence of multicollinearity. VIF values ranging from 1 to 10 indicate the absence of multicollinearity. The effect size values were interpreted as small (η^2^ = 0.01, r = 0.10, V = 0.10), medium (η^2^ = 0.06, r = 0.30, V = 0.30), or large (η^2^ = 0.14, r = 0.50, V = 0.5) [41]. A value of *p* < 0.05 was considered statistically significant. All statistical analyses were performed using IBM SPSS Statistics, version 28.0 (IBM Corp., Armonk, NY, USA).

### 2.6. Ethical Considerations

The study adhered to the principles outlined in the Declaration of Helsinki and Ethical Guidelines for Medical and Health Research Involving Human Subjects. The requirement for written informed consent was waived because of the retrospective study design. Instead, an opt-out policy was adopted, which was disclosed on the hospital website. The patients were allowed to withdraw from the study at any time. This study was approved by the Ethics Committee of Ibaraki Prefectural University of Health Sciences (approval number: e422; approval date: 21 December 2023).

## 3. Results

### 3.1. Participants

The baseline characteristics of the study participants are shown in Table 1. In total, 62 participants were included. The mean age of all participants was 78.2 ± 8.3 years, and 75.8% were women. Injury causes included 83.9% falls to the ground, 6.5% falls from a height, and 9.7% other causes. Fracture types included neck (50.0%), trochanteric (41.9%), and subtrochanteric (8.1 %) fractures. Surgery types included 62.9% open reduction and internal fixation, 6.5% total hip arthroplasty, and 30.6% bipolar hip arthroplasty.

Sarcopenia was diagnosed in 15 (24.2%) participants, who were older (82.9 ± 8.4 vs. 76.7 ± 7.8 years, respectively, *p* = 0.001) and included a lower proportion of women (46.7 vs. 85.1%, *p* = 0.005) than did participants without sarcopenia. Injury causes, fracture type, and surgery type were not significantly different between participants with and without sarcopenia. The surgery-to-DXA-scan duration was significantly longer for participants with sarcopenia than for those without (31.0 [21.0–42.0] vs. 22.0 [19.0–28.0] days, respectively, *p* = 0.021), indicating that patients with sarcopenia stayed longer in the acute hospital postoperatively than did those without sarcopenia. The CCI score was higher for patients with sarcopenia than for those without (2.0 [1.0–3.0] vs. 1.0 [0.0–2.0] points, respectively, *p* = 0.048). Comorbid neurological conditions were observed in 15 (24.2%) participants: four participants in the sarcopenia group and eight in the non-sarcopenia group had chronic cerebrovascular diseases; two participants in the non-sarcopenia group had Parkinson’s disease; and one participant in the non-sarcopenia group had peroneal nerve palsy. The prevalence of comorbid neurological conditions was not significantly different between groups (26.7% vs. 23.4%, *p* = 0.523). Although patients with sarcopenia had lower FIM motor (58.0 [45.0–64.0] vs. 64.0 [52.0, 71.0] points, respectively) and cognition (30.0 [24.0–32.0] vs. 31.0 [27.0–32.0] points, respectively) scores than did those without sarcopenia, the difference was not statistically significant (*p* = 0.054 and *p* = 0.402, respectively). All patients were independent ambulators (FAC > 3) before the injury. Anthropometric measures, including body height, weight, BMI, handgrip strength, and SMI, were not significantly different between participants with and without sarcopenia. 

### 3.2. Functional Outcomes

Functional outcomes at the time of discharge are presented in Table 2. The mean hospitalization duration, overall home discharge rate, and median daily rehabilitation duration were 65.9 ± 17.6 days, 95.2%, and 2.4 (2.3–2.5) h, respectively; these variables showed no significant differences between patients with sarcopenia and those without sarcopenia (*p* = 0.511, *p* = 0.571, and *p* = 0.593, respectively). BBS scores at discharge were significantly lower for patients with sarcopenia than for those without (41 [29.0–49.0] vs. 49 [42.0–55.0] points; *p* = 0.004), while the prevalence of poor balance function (as defined by BBS ≤45 points) at the time of discharge was higher among patients with sarcopenia than among those without (73.3 vs. 40.4%, respectively, *p* = 0.038). The number of patients who could walk independently (as defined by FAC > 3 points) at the time of discharge was not significantly different between groups (86.7% vs. 95.7%, *p* = 0.244). The self-selected walking speed at discharge (0.8 ± 0.3 vs. 1.0 ± 0.4 m/s; *p* = 0.029) and the FIM motor and total scores (74.0 [61.0–80.0] vs. 81.0 [79.0–84.0] points, respectively, *p* = 0.008; 104.0 [92.0–113.0] vs. 113 [106.0, 117.0] points, *p* = 0.100) were significantly lower for patients with sarcopenia than for those without sarcopenia. Although the median FIM cognition score was lower for patients with sarcopenia than for those without (32.0 [28.0–33.0] vs. 33.0 [30.0–34.0] points, respectively), the difference was not significant (*p* = 0.073).

### 3.3. Association between Sarcopenia and Balance Function

The results of the multivariate linear regression analysis are presented in Table 3. No multicollinearity was observed among the variables. The results revealed that baseline sarcopenia was independently associated with the BBS score at discharge (β = −0.283, *p* = 0.038). Age, sex, comorbidities (assessed using the CCI), and cognitive function (assessed using the FIM cognition score) were not significantly associated with the BBS score at discharge.

## 4. Discussion

To the best of our knowledge, this is the first study to examine the impact of sarcopenia on balance outcomes in patients who underwent inpatient rehabilitation after hip fracture surgery. The findings revealed that baseline sarcopenia is independently associated with poor balance outcomes at discharge.

Sarcopenia is associated with poor balance. This association has been previously reported in community-dwelling individuals with sarcopenia, with a higher incidence of falls than that in individuals without sarcopenia [19,20,21,42]. However, information regarding the possible association between sarcopenia and balance outcomes in patients undergoing inpatient rehabilitation is limited. Recently, Lim et al. conducted a two-week fragility fracture-integrated rehabilitation program for patients with or without sarcopenia following hip fracture surgery and compared the changes in their functional levels [37]. Their results showed that patients with sarcopenia had significantly lower BBS scores than did those without sarcopenia, both before and after the program, indicating that patients with sarcopenia had inferior balance function compared with that shown by individuals without sarcopenia. Our results align with this finding, as patients with sarcopenia exhibited significantly lower BBS scores than did those without sarcopenia. Additionally, a higher proportion of patients with poor balance function, as indicated by a BBS of ≤45 points, was found in the sarcopenia group compared with that in the non-sarcopenia group. Furthermore, multivariate regression analysis adjusted for age, sex, comorbidities, and cognitive function revealed a negative association between sarcopenia and balance. These results suggest that patients with sarcopenia are more likely to have persistent balance problems than are those without sarcopenia after inpatient rehabilitation.

The mechanisms underlying the relationships between sarcopenia, balance, and fractures are complex. Maintaining balance requires coordination of the motor, nervous, and sensory systems [19,21]. Age-related decline in these functions may explain the link between sarcopenia, poor balance, and an increased risk of falls [21,39,42]. Additionally, sarcopenia is associated with osteoporosis [43], aggravating the risk factors for subsequent fractures and poor balance [44]. Furthermore, falls and fractures can lead to reduced mobility, balance deficits, and fear of falling [25]. Physical inactivity stemming from hospitalization, ADL dependency, and poor diet can exacerbate the loss of muscle mass and strength, aggravate sarcopenia, and further increase the risk of poor balance, falls, and secondary fractures [45]. Sarcopenia, balance issues, and fractures mutually influence each other, with one possibly leading to the other.

Patients with sarcopenia may have poor balance function and exhibit lower levels of ADL than do those without sarcopenia. In our study, FIM motor and total scores were significantly lower for patients with sarcopenia than for those without at discharge. This result is consistent with that reported by Landi et al., who demonstrated that after hip fractures, patients with sarcopenia had lower levels of ADL (as assessed by Barthel index scores) at the time of discharge and at the three-month follow-up examination compared with those without sarcopenia [11]. In another study, Kanaya et al. showed that patients with sarcopenia had significantly lower FIM total, motor, and cognitive scores than did those without sarcopenia [15]. Furthermore, studies that examined the association between sarcopenia and functional outcomes reported a negative association between sarcopenia and ADL in inpatient rehabilitation settings [10,46]. As balance function is closely associated with ADL independence [47,48], persistent balance deficits may negatively affect ADL, especially in individuals with sarcopenia.

The relationship between sarcopenia and poor functional outcomes after postoperative inpatient rehabilitation is noteworthy because sarcopenia is prevalent in patients with hip fractures. The reported prevalence of sarcopenia varies depending on the population, diagnostic tools, and definition of sarcopenia [12,14,15,22,23]. In this study, the prevalence of sarcopenia was 24.2%. In similar in-hospital rehabilitation wards, the prevalence has been reported to be as high as 60% [12,15]. One possible explanation for this difference is a discrepancy in the diagnostic tools used. Yoshimura et al. used bioelectrical impedance analysis (BIA), whereas we used DXA for skeletal muscle mass assessment. BIA reportedly underestimates fat-free mass, especially in patients with a lower BMI [49]. Additionally, the prevalence of sarcopenia varies substantially depending on the ASM indices used to define sarcopenia [23]. Previous studies have defined SMI by adjusting ASM according to the square of height; whereas in our study, we defined SMI by adjusting ASM according to BMI. The latter method is suggested by the AWGS for diagnosing sarcopenia when DXA is used for skeletal muscle mass assessment [17] and has been reported to predict better functional outcomes [38]. Furthermore, differences in sample size and patient characteristics, such as age, sex ratio, number of comorbidities, pre-injury activity levels, and differences in the surgery-to-DXA-scan duration may explain the differences in the reported prevalence of sarcopenia between studies.

Regardless of the differences in the reported prevalence of sarcopenia, it is a prevalent condition in patients with hip fractures. Its negative association with functional outcomes highlights the significance of timely recognition for early intervention. Several variables have been recognized as potential risk factors for sarcopenia in patients with hip fracture. In a systematic review, Chiang et al. identified factors predicting sarcopenia, including advanced age, male sex, low BMI, cognitive dysfunction, insufficient preinjury activity, low handgrip strength, and severe osteoporosis [3]. Our findings are consistent with the previous reports, as patients with sarcopenia were older and had a higher proportion of male participants; however, BMI, cognitive function, preinjury activity, and hand grip strength were not statistically different between the groups, possibly due to differences in patient characteristics, assessment tools, and sample sizes. A longer duration from surgery-to-DXA scan was recorded for patients with sarcopenia than for those without, supporting the findings of studies reporting an association between sarcopenia and a longer hospitalization duration in acute hospitals [50]. This could be attributed to the fact that patients with sarcopenia experience more postsurgical complications, require more time until their medical condition stabilizes, and are often referred to rehabilitation wards. By leveraging these variables as indicators, patients with sarcopenia should be promptly recognized and appropriate management programs should be implemented to mitigate the aggravation of sarcopenia, improve balance and related functional outcomes, and reduce the risk of recurrent falls and fractures.

Effective strategies for managing sarcopenia and enhancing functional outcomes should incorporate physical exercises with proper nutrition. Resistance training has been recommended as an effective means to increase muscle mass and function in individuals with sarcopenia [51]. In addition, the benefits of resistance training may be enhanced by nutritional interventions, such as ensuring adequate protein and calorie intake [52]. Moreover, task-specific balance training should be incorporated to improve balance. Postoperative rehabilitation programs commonly include range-of-motion exercises, muscle strengthening exercises, standing and gait exercises with weight-bearing progression in the affected lower limb, and stair-climbing exercises [24]. Studies have shown that these types of exercises improve functional independence in the ADLs and gait ability [53,54]. However, these programs may not effectively reduce the long-term risks of falls and refractures [24]. Instead, fall prevention programs and balance task-specific training have been reported to be more effective than conventional motor rehabilitation in enhancing balance, lower limb strength, daily activities, and overall quality of life in older individuals recovering from hip fractures [55,56]. Therefore, balance-specific training should be considered, in conjunction with resistance training and nutrition, particularly for patients with sarcopenia.

This study has some limitations. First, it was a retrospective cohort study conducted at a single local rehabilitation hospital and had a relatively small sample size, which limited the generalizability of our results. Second, we included participants with neurological comorbidities, such as chronic cerebral vascular diseases and Parkinson’s disease, because these comorbidities are common in patients who are referred to rehabilitation hospitals after hip fracture surgery. Although the prevalence of neurological comorbidities was not significantly different between patients with and without sarcopenia, these conditions may have influenced our results. Third, we included patients who underwent inpatient rehabilitation during the coronavirus disease 2019 pandemic, which may have influenced the progress and outcomes of their rehabilitation. Nevertheless, we ensured the continuous provision of inpatient rehabilitation services throughout this period; thus, this effect may be minimal. Fourth, sarcopenia was assessed upon admission. Given the retrospective nature of the study, the sarcopenia status before surgery could not be determined. In addition, muscle mass and SMI have been reported to decrease after hip fracture surgery [45], particularly in patients with sarcopenia [57]. Therefore, the sarcopenia status before surgery and changes in the body composition after surgery and during inpatient rehabilitation may have influenced these results. Fifth, details of pre-fracture ambulatory and cognitive functions could not be obtained because of the retrospective nature of this study. Pre-fracture ambulatory and cognitive functions may be related to functional recovery and balance [7,40]; therefore, future studies should incorporate this information into a prospective design.

Despite these limitations, our study demonstrated that patients with baseline sarcopenia may struggle with balance deficits after inpatient rehabilitation, which may negatively affect their ADLs and their quality of life. Therefore, healthcare professionals should promptly diagnose sarcopenia as a modifiable risk factor. Resistance training and nutritional management should be incorporated into sarcopenia treatment. Moreover, a more suitable rehabilitation program, including balance task-specific training, should be designed for patients with sarcopenia to improve balance function [21,47]. Nevertheless, no evidence has shown that the treatment of sarcopenia improves these outcomes [3]. Further research is needed to investigate the relationship between sarcopenia and balance, and to examine the impact of sarcopenia treatment on these outcomes.

## 5. Conclusions

After inpatient rehabilitation, patients with baseline sarcopenia had inferior balance outcomes compared to those without sarcopenia at discharge. Sarcopenia should be assessed on admission to consider and provide additional care for those with a higher risk of poor functional outcomes. More studies are needed to investigate the association between sarcopenia and functional outcomes, examine the impact of sarcopenia treatment on these outcomes, and reduce the risk of recurrent falls and fractures in patients with hip fractures.

## Figures and Tables

**Table 1:** Baseline characteristics of the participants.

	Total (*n* = 62)		Sarcopenia (*n* = 15)		Non-Sarcopenia (*n* = 47)		*p*	Effect Size	
Age	78.2	±8.3	82.9	±8.4	76.7	±7.8	0.010 *	0.79	a
Sex, number of women	47	75.8%	7	46.7%	40	85.1%	0.005 *	0.38	c
Cause of Injury									
Falls to the ground	52	83.9%	13	86.7%	39	83.0%	0.589	0.16	c
Falls from a height	4	6.5%	0	0.0%	4	8.5%			
Others	6	9.7%	2	40.0%	4	8.5%			
Fracture Type									
Neck fracture	31	50.0%	7	46.7%	24	51.1%	0.902	0.06	c
Trochanteric fracture	26	41.9%	7	46.7%	19	40.4%			
Subtrochanteric fracture	5	8.1%	1	6.7%	4	8.5%			
Surgery type									
ORIF	39	62.9%	8	53.3%	31	66.0%	0.709	0.12	c
THA	4	6.5%	1	6.7%	3	6.4%			
BHA	19	30.6%	6	40.0%	13	27.7%			
Surgery-to-DXA-scan duration (days)	23.5	(19.0–32.3)	31.0	(21.0–42.0)	22.0	(19.0–28.0)	0.021 *	0.29	b
CCI	1.0	(0.0–2.0)	2.0	(1.0–3.0)	1.0	(0.0–2.0)	0.048 *	0.25	b
Comorbid neurological conditions	15	24.2%	4	26.7%	11	23.4%	0.523	0.03	c
Independent ambulators before fracture (FAC > 3)	62	100%	15	100%	47	100%	–	–	–
FIM									
Motor score	61.5	(48.8–70.0)	58.0	(45.0–64.0)	64.0	(52.0–71.0)	0.054	0.25	b
Cognition score	30.0	(27.0–32.0)	30.0	(24.0–32.0)	31.0	(27.0–32.0)	0.402	0.11	b
Total score	90.0	(75.5–99.25)	82.0	(68.0–94.0)	92.0	(76.0–102.0)	0.084	0.22	b
Anthropometric measures									
Body height (cm)	154.4	±8.4	156.0	±8.2	153.9	±8.4	0.399	0.25	a
Body weight (kg)	51.6	±10.4	53.1	±8.3	51.1	±11.1	0.519	0.19	a
BMI (weight/height^2^)	21.6	±3.6	21.9	±3.6	21.5	±3.7	0.682	0.12	a
Handgrip strength (kg)	18.3	(14.0–20.6)	15.4	(14.0–23.0)	18.5	(14.0–20.0)	0.780	0.04	b
SMI adjusted by height^2^	5.4	(4.9–6.0)	5.1	±0.7	5.6	±0.9	0.064	0.56	a
SMI adjusted by BMI	0.6	(0.5–0.7)	0.6	(0.5–0.7)	0.6	(0.6–0.7)	0.233	0.15	b

The values are presented as *n* (%), mean ± SD, or median (IQR). Abbreviations: BHA, bipolar hip arthroplasty; BMI, body mass index; CCI, Charlson Comorbidity Index; FIM, Functional Independent Measure; IQR, interquartile range; *n*, number; ORIF, open reduction and internal fixation; SD, standard deviation; SMI, skeletal muscle index; THA, total hip arthroplasty. a: *t*-test, Cohen’s d; b: Mann–Whitney U test, Cramer’s V; c: Fisher’s exact test, effect size r. * *p* < 0.05.

**Table 2:** Functional outcomes at the time of discharge.

		Total (*n* = 62)		Sarcopenia (*n* = 15)		Non-Sarcopenia (*n* = 47)		*p*	Effect Size	
Length of hospital stay (days)		65.9	±17.6	68.0	(51.0–84.0)	65.0	(54.0–80.0)	0.511	0.08	b
Number of home discharge		59	95.2%	14	93.3%	45	95.7%	0.571	0.05	d
Daily rehabilitation therapy (hours)		2.4	(2.3–2.5)	2.4	(2.3–2.5)	2.4	(2.2–2.5)	0.593	0.07	b
BBS score		47.0	(40.5–54.3)	41.0	(29.0–49.0)	49.0	(42.0–55.0)	0.004 *	0.37	b
Poor balance (BBS ≤ 45)		30	48.4%	11	73.3%	19	40.4%	0.038 *	0.28	c
FAC										
	≤3	4	6.5%	2	13.3%	2	4.3%	0.255	0.18	d
	4	10	16.1%	3	20.0%	7	14.9%			
	5	48	77.4%	10	66.7%	38	80.9%			
Independent ambulators (FAC > 3)		58	93.5%	13	86.7%	45	95.7%	0.244	0.21	d
SWS (m/s)		1.0	±0.4	0.8	±0.3	1.0	±0.4	0.029 *	0.66	a
FIM										
	Motor score	80.0	(74.0–83.3)	74.0	(61.0–80.0)	81.0	(79.0–84.0)	0.008 *	0.34	b
	Cognition score	32.0	(30.0–34.0)	32.0	(28.0–33.0)	33.0	(30.0–34.0)	0.073	0.23	b
	Total score	112.0	(103.3–116.0)	104.0	(92.0–113.0)	113.0	(106.0–117.0)	0.010 *	0.33	b

The values are presented as *n* (%), mean ± SD, or median (IQR). Abbreviations: BBS, Berg Balance Scale; FAC, functional ambulation category; FIM, functional independence measure; IQR, interquartile range; *n*, number; SD, standard deviation; SWS, self-selected walking speed. a: *t*-test, Cohen’s d; b: Mann–Whitney U test, Cramer V; c: chi-square test, effect size r; d: Fisher’s exact test, effect size r. * *p* < 0.05.

**Table 3:** Multivariate linear regression analysis of the BBS score at the time of discharge.

	BBS Score at the Time of Discharge			
	Standardized Coefficient			
	β	(95% CI)	*p*	VIF
Constant		(32.015–92.744)	<0.001 *	
Age	−0.248	(−0.596–0.005)	0.054	1.252
Sex (women/men)	0.109	(−3.454–8.447)	0.404	1.330
CCI	−0.128	(−3.08–0.972)	0.302	1.192
FIM cognition score at admission	0.216	(−0.041–0.908)	0.073	1.105
Sarcopenia (non-sarcopenia/sarcopenia)	−0.282	(−12.592–[−0.382])	0.038 *	1.400

Abbreviations: BBS, Berg Balance Scale; CCI, Charlson Comorbidity Index; FIM, Functional Independent Measure; VIF, variation inflation factor. * *p* < 0.05.

## Data Availability

No new data were created or analyzed in this study.
